# Phosphate-Solubilizing *Bacillus* sp. Modulate Soil Exoenzyme Activities and Improve Wheat Growth

**DOI:** 10.1007/s00248-023-02340-5

**Published:** 2024-01-16

**Authors:** Zafar Iqbal, Maqshoof Ahmad, Muhammad Ali Raza, Thomas Hilger, Frank Rasche

**Affiliations:** 1 National Research Center of Intercropping, The Islamia University of Bahawalpur, Bahawalpur, 63100 Pakistan; 2 Department of Soil Science, The Islamia University of Bahawalpur, Bahawalpur, 63100 Pakistan; 3 Faculty of Agricultural Sciences, Institute of Agricultural Sciences in Tropics (Hans-Ruthenberg-Institute), University of Hohenheim, 70593 Stuttgart, Germany; 4 Ghansu Academy of Agricultural Sciences, Lanzhou, 730070 Gansu China; 5 International Institute of Tropical Agriculture (IITA), PO Box 30772-00100, Nairobi, Kenya

**Keywords:** *Bacillus* sp., Phosphate solubilization, Mineralization, Phosphate esterase, ß-D glucosidase

## Abstract

Phosphorus (P) is a vital mineral nutrient in agriculture and its deficiency results in reduced growth, yield, and grain quality in cereals. Much of the applied P in agriculture becomes fixed in soils, limiting its accessibility to plants. Thus, investigating sustainable strategies to release fixed P resources and enhance plant uptake is crucial. This study explored how plant-associated bacteria employ phosphate solubilizing mechanisms to improve P availability. The growth patterns of four bacterial strains, namely *Bacillus subtilis* ZE15 and ZR3, along with *Bacillus megaterium* ZE32 and ZR19, were examined in Pikovskaya’s broth culture with and without the addition of insoluble phosphorus (P). In the absence of P amendment, most strains reached a stationary growth phase by the fourth day. However, their responses diverged when exposed to P-amended media. Particularly, ZE15 demonstrated the highest P solubilization capability, achieving up to 130 µg mL^−1^ solubilization in vitro. All strains produced organic acids in Pikovskaya’s broth culture. A comparison of the influence of Ca_3_(PO_4_)_2_ revealed significantly greater organic acid quantities in the presence of insoluble P. Notably, strain ZE15 exhibited the highest phosphate esterase activity (3.65 nmol g^−1^ dry matter), while strain ZE32 showed the highest ß-D glucosidase activity (2.81 nmol g^−1^ dry matter) in the presence of insoluble P. The ability of *Bacillus* species to solubilize P in combination with increased exoenzyme activity in the rhizosphere could be used in future studies to support P uptake through enhanced solubilization and mineralization.

**Supplementary Information:**

The online version contains supplementary material available at 10.1007/s00248-023-02340-5.

## Introduction

Phosphorus (P) availability is a critical restraint on plant development in agriculture, attributed to its vital roles in plant structure, function, and metabolism [1]. Plants contain approximately 0.8% phosphorus in their dry matter composition [2], and deficiency symptoms can occur when P concentration in leaves is below 0.2% [3]. About 75% of applied phosphatic fertilizers remain fixed in soil [4], which depends largely on soil pH [5]. Historically, increasing amounts of P fertilizers have been applied to agricultural soils to fulfill the P needs of the plants, with acknowledged hazardous environmental effects [6]. Solution-oriented nutrient management practices are needed to improve P use efficiency [6].

One promising approach that has gained special attention in the recent past is the use of phosphate-solubilizing microorganisms (PSM) to improve the bioavailability and uptake of P to support plant growth [7]. This group of bacteria has gained attention to complement sustainable agricultural practices [8], whereby they have been verified to promote plant growth under deficient P conditions. PSMs function efficiently under P stress, while their phosphate solubilizing ability is inhibited in the presence of soluble fertilizers [9]. Using an array of different mechanisms [10], several PSMs (e.g. *Bacillus*, *Pseudomonas, Rhizobium*) can produce growth regulators, such as indole-3-acetic acid (IAA) as a direct mechanism [11]. In addition, rhizobacteria (e.g., *B. subtilis*, *B. megaterium*, *B. aryabhattai*) with the ability to solubilize P were verified to produce auxin and ACC-deaminase, resulting in root growth promotion [12]. Indirect mechanisms facilitate the availability of nutrients through microbially synthesized organic acids and enzymes that solubilize endogenous and insoluble P in soils [13]. Specifically, microbial alkaline phosphatase (ALP) and acid phosphatase (ACP) stimulate the secretion of organic acids that improve P uptake as a function of increasing the availability of solubilized P in soil solution [14]. In the process of organic acid-mediated P solubilization, involved bacteria deplete the rhizosphere pH, chelate cations, and compete for adsorption sites in the soil. It has also been reported that organic acids form a complex with metal ions and release P [15]. Additionally, exoenzymes, diverse in targeting organic compounds, exhibit evolutionary variations. Isozymes, despite catalyzing the same reactions, differ significantly in kinetic properties like turnover rate (*k*_cat_), maximal velocity (*V*_max_), Michaelis constant (*K*_m_, inversely linked to substrate affinity), and catalytic efficiency (*k*_cat_/*K*_m_). In soils, exoenzyme kinetics reflect nutrient availability, climate, pH, and proximity to plant roots. Microbe-plant interactions, soil characteristics, and environmental conditions collectively influence enzyme expression, turnover, mobility, and substrate accessibility, highlighting the intricacies of microbial ecology.

It is well-described that microbial application can increase P solubilization; however, the underlying relationship between bacterial growth, pH depletion, and solubilized P remains elusive. Whereby this relationship is yet to be verified, it could also be assumed that plant growth–promoting bacteria have the ability to produce organic acids that enable the solubilization of P from insoluble sources. Hence, the present study aims to quantify the production of organic acids of the respective bacterial strains and measure the levels of solubilized P and pH depletion with and without insoluble P in broth culture. Furthermore, the in vitro results verify in terms of soil exoenzyme activities which improve plant growth under P deficiency. It is expected that the mechanisms underlying phosphate solubilization by the *Bacillus* strain through the production of organic acids and soil exoenzyme activities and the potential application of PSB will promote plant growth.

## Materials and Methods

### Selection of Strains

Pre-identified soil–plant-associated bacterial strains *Bacillus subtilis* ZE15 and ZR3 and *Bacillus megaterium* ZE32 and ZR19 were evaluated for growth diversity at a wide range of pH, secretion of organic acids, and plant growth promotion [16].

### Quantitative Analysis of Solubilized Phosphorous

The quantitative analysis of phosphorus (P) solubilization was carried out following the procedure outlined by Clesceri et al. [17]. For the assessment of solubilized P, 10 ml of Pickovskaya’s broth was taken into falcon tubes and inoculated with 20 µL of bacterial culture having an optical density (OD) of 0.6 at 600 nm. The tubes were then placed in an incubator set at 30 ± 1 °C with continuous agitation. On a daily basis, the culture tubes were subjected to centrifugation, and a microwell plate was utilized to take 150 µL of the resulting liquid, followed by adding an equivalent volume of vanadomolybdate solution. This mixture was then subjected to incubation for 1 h to allow for the development of a yellow color. Subsequently, the absorbance of the solution was measured at a wavelength of 410 nm using a microplate reader (Nanoquant Infinite 200pro, Tecan, Männedorf, Switzerland). Similarly, a calibration curve was generated with standard solutions having concentrations of 20, 40, 60, 80, 100, 120, and 140 mg L^−1^ P. The concentration of P was calculated by comparing the sample readings with the standard calibration curve.

### Growth of Strains at Various pH Levels

The bacterial strain’s growth was assessed across various pH levels, spanning from acidic to alkaline conditions. To achieve acidity (pH 5.0 and 6.0), hydrochloric acid (1 M) was used, while alkalinity (pH 8.0 and 9.0) was attained using sodium hydroxide (1 M). A reference medium of pH 7.0 was also prepared. Bacterial cultures (5 µL) with 1.0 OD_600_ were introduced into 295 µL of sterilized nutrient broth separately in a microwell plate for respective pH levels. The cultures were then incubated at a temperature of 30 ± 1 °C in a rotary shaker at 125 rpm. The microplate reader was used to measure OD at 600 nm at 30-min intervals.

### Determination of Organic Acids by Bacterial Strains

Bacterial cultures were assessed for the production of organic acids in nutrient media using the extraction and methylation technique, as outlined in the study by Politz et al. [18]. To execute the experiment, Falcon tubes were filled with 19.90 mL of sterilized Pickovskaya’s media. These tubes were inoculated with 100 µL of bacterial culture with an optical density (OD) of 1.0 at 600 nm, and the media were prepared both with and without Ca_3_(PO_4_)_2_. Subsequently, the tubes were wrapped using parafilm and subjected to incubation at 30 ± 1 °C temperature. The incubation occurred with continuous shaking in a rotary shaker at 125 rpm for 10 days. Following incubation, the representative bacterial culture (3 mL) was transferred into a test tube. Then, in each tube, 15 µL of internal standard I was added, which comprised dichloro undecanoic acid (1.77 mg) dissolved in 1 mL of hexane. Additionally, 100 µL of glacial acetic acid and 5 mL of a 1:1 mixture of chloroform and methanol were introduced to each tube. These tubes were precisely vortexed and subsequently centrifuged for 10 min at 4000 rpm. The resulting lower aqueous layer was isolated using a separation funnel. The chloroform component was evaporated using a nitrogen stream until dry residues remained within the tube.

For the methylation process, 1 mL of sulfuric methanol (1%) solution was incorporated into the identical tube. The samples were then heated at 80 °C for 1 h. Following this, samples were cooled using ice and subjected to sequential treatment with 2 mL of n-hexane, followed by 1 mL of saturated sodium chloride, and 2 mL of sterilized distilled water. Rigorous agitation was performed, and the resulting upper organic layer was separated. This organic layer was combined with 15 µL of internal standard II (comprising 0.5 mg of myristic acid ethyl ester in 1 mL of hexane) before being transferred into a gas chromatography [9] vial. This prepared mixture was then subjected to GC–MS analysis.

The gas chromatography analysis was conducted using an Agilent 6890 GC system equipped with a flame ionization detector (FID) from Agilent Technologies Korea in Seoul, South Korea. The Chemstation Software, also from Agilent Technologies Korea, was employed for instrument control and data processing. The capillary column employed for the analysis was an AT-1000 (0.53 mm I.D. × 15 mL, 1.2 µm df) obtained from Alltech in Illinois, USA. To determine the organic acids, 2 µl of the bacterial sample under consideration was injected into the system. The following gas chromatography conditions were applied: Helium served as the carrier gas, the initial column temperature was set at 50 °C and held for 1 min, then increased to 125 °C at a rate of 25 °C/min, with further elevation to 300 °C at 10 °C/min, and a subsequent 15-min hold. The injection temperature was maintained at 250 °C, using a split injection mode, a gas flow of 1.7 ml/min in the column, and employing the analytical column DB-5 ms. For mass spectrometry (MS) analysis, the ion source temperature was established at 200 °C, the interface temperature at 280 °C, a solvent cut time of 3 min, and a detector gain mode set to relative.

### Evaluation of Strains Under Axenic Conditions for Promotion of Plant Growth

The plant growth–promoting traits of the bacterial strains were assessed through a rhizobox experiment. To carry out this experiment, sanitized rhizoboxes with dimensions of 6 cm in length, 2.5 cm in width, and 20 cm in height were utilized. The rhizobox was constructed from plastic with a transparent and removable side, allowing for the observation of root growth and the collection of samples without causing harm to the roots and the surrounding rhizosphere. These boxes were filled with 200 g of sterilized sand. The seeds of the wheat (*Triticum aestivum*) variety Johar-2016 were subjected to surface sterilization. This process involved immersing the seeds in 95% ethanol for 30 s, followed by a 3-min dip in a 0.2% solution of mercuric chloride (HgCl_2_). Subsequently, the seeds were thoroughly rinsed with sterile distilled water. Inoculation of the bacterial strains to the seeds was accomplished by immersing them in the respective broth culture for 30 min. The sanitized broth was used to prepare un-inoculated control seeds. These treated seeds were then planted in the rhizoboxes. To meet the nutritional requirements of the seedlings, half of the rhizoboxes were irrigated using a sterilized, half-strength Hoagland solution [19]. Meanwhile, the remaining half was treated with the same solution but with the inclusion of 2.5 g L^−1^ of tricalcium phosphate (Ca_3_(PO_4_)_2_), serving as a substitute for potassium dihydrogen phosphate (KH_2_PO_4_). The arrangement of the rhizoboxes followed a randomized complete factorial design and included three replications. The entire setup was located within a growth chamber under the following specific conditions: a daytime temperature of 20 ± 1 °C, a nighttime temperature of 15 ± 1 °C, a humidity level of 70%, and a light intensity ranging from 1400 to 1500 µmol m^−2^ s^−1^, with a 12-h cycle of light and darkness. After a period of 30 days, the dry biomass of the seedlings was measured. Furthermore, we performed measurements on root attributes such as length, diameter, and volume utilizing a root scanner (Expression 11000XL, Epson, Amsterdam, Netherlands). To evaluate microbial activity within the rhizosphere, we employed techniques for assessing phosphate esterase and ß-D-glucosidase activities as per the guidelines detailed in the work of Hendriksen et al. [20].

### Statistical analyses

Recorded data were expressed as means of three replicates. For statistical analysis, a linear model with a completely randomized design (CRD) was utilized using one-way analysis of variance. Statistics 8.1 software package (Statistix®, Analytical Software, Tallahassee, FL, USA) was used to do multiple means comparisons at the 5% level of confidence [21]. Organic acids were plotted in multidimensional scaling (MDS) using the R project for statistical computing [22]. Origin 2018 (OriginLab Corporation, Northampton, USA) was used to conduct a linear regression and correlation analysis.

## Results

### Growth Behavior and Potential Effect of Strains on pH and Solubilized P in Culture Media

Bacterial growth was observed in both the standard Pickovskaya’s nutrient broth and in the cultures enriched with an insoluble phosphorus source (Ca_3_(PO_4_)_2_). The broth cultures exhibited notable diversity in bacterial growth patterns (Supplementary Fig. 1). Findings indicated that bacterial strains entered the stationary phase by the fourth day of incubation in the absence of Ca_3_(PO_4_)_2_. Conversely, the presence of Ca_3_(PO_4_)_2_ stimulated distinct behaviors in these strains. Notably, *Bacillus subtilis* (ZE15) entered the stationary phase, achieving the highest OD of 0.77 on the sixth day. On the fifth day of incubation, strains ZE32, ZR3, and ZR19 reached the stationary phase with optical densities (OD_600_) of 0.58, 0.75, and 0.70, respectively. When exposed to phosphorus stress, these strains exhibited divergent growth patterns. Initially, they showed resistant growth; however, beyond the third day of incubation, the bacterial strains acclimated, leading to rapid growth. At seventh day of incubation, the strains ZE15, ZR3, ZE32, ZR3, and ZR19 exhibited maximum growth, with bacterial counts reaching 1.11 × 10^6^, 1.06 × 10^6^, 9.64 × 10^5^, and 9.06 × 10^105^ CFU mL^−1^, respectively.

**Fig. 1:**
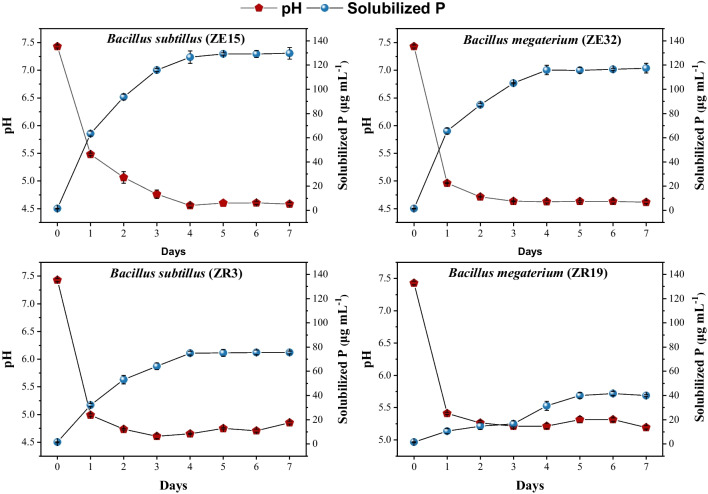
Effect of plant-associated bacteria on P solubilization and pH of growth media. A reverse relation was found between solubilized P contents and the pH of the broth culture

The impact of bacterial strains on pH and solubilized phosphorus content in Pickovskay’s broth culture was depicted in Fig. 1. The solubilized phosphorus content was detected until the seventh day of incubation. The strain ZE15 demonstrated the highest level of solubilized phosphorus, measuring 130 µg mL^−1^ after 5 days of incubation. In comparison, strains ZE32 and ZR3 yielded 117 µg mL^−1^ and 76 µg mL^−1^ of solubilized phosphorus, respectively, during the same incubation period. Strain ZR19 displayed the lowest solubilized phosphorus content (42 µg mL^−1^). Additionally, the pH of the broth culture was monitored and demonstrated a declining trend over time. Strain ZE15 exhibited the most significant decline in pH (− 38%), correlating with the highest solubilized phosphorus levels.

### The Correlation Among Bacterial Growth, pH Levels, and Solubilization of P

**Fig. 2:**
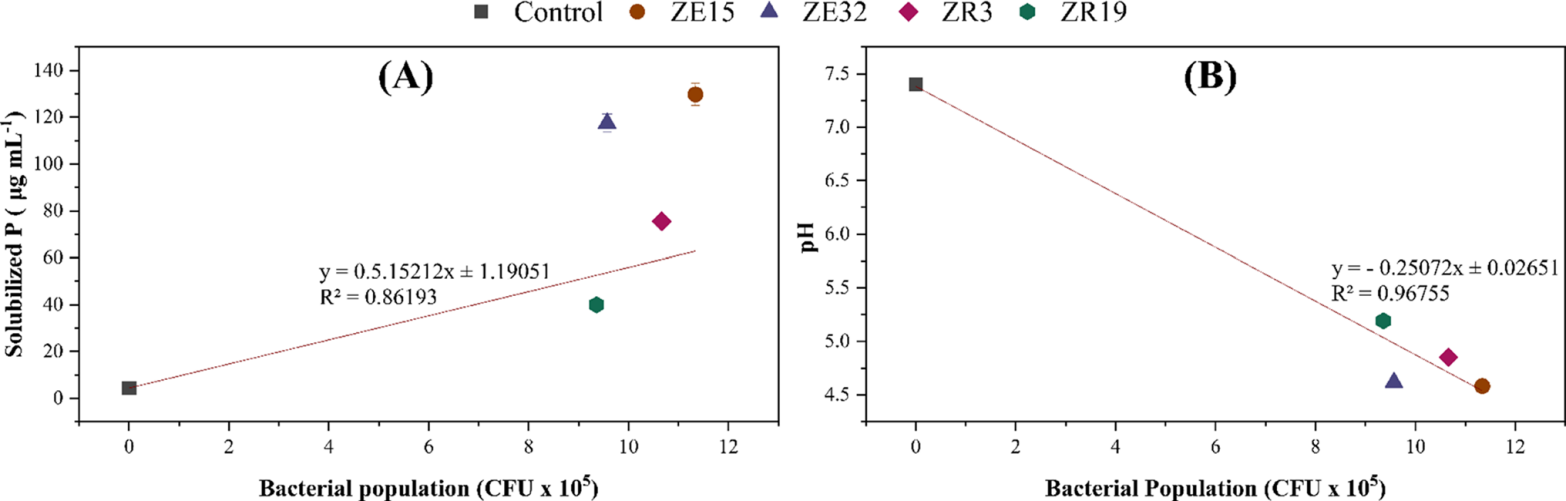
The bacterial population plays a crucial role in P solubilizing activities. (**A**) Indicated a direct connection between the bacterial population and the solubilized P in the growth medium. **(B)** Demonstrated an inverse relationship between the bacterial population and the pH of the growth medium

The relationship between pH and solubilized P and the bacterial population, quantified as colony-forming units (CFU) in the growth medium is presented in Fig. 2, indicating that the bacterial population was directly proportional to solubilized P content. Tested bacterial strains exhibited improved performance in the presence of insoluble P (calcium phosphate, Ca_3_(PO_4_)_2_). Furthermore, an inverse relationship was observed between the bacterial population and the pH of the broth culture. A negative value of correlation coefficient (*R* =  − 0.98, *p* ≤ 0.0017) indicated a decline in pH with increasing bacterial population. A positive correlation (*R* = 0.79) was observed between bacterial population and solubilized P content.

### Quantification of Organic Acids in Pikovskaya’s Broth Culture with and Without Insoluble Phosphorus

**Fig. 3:**
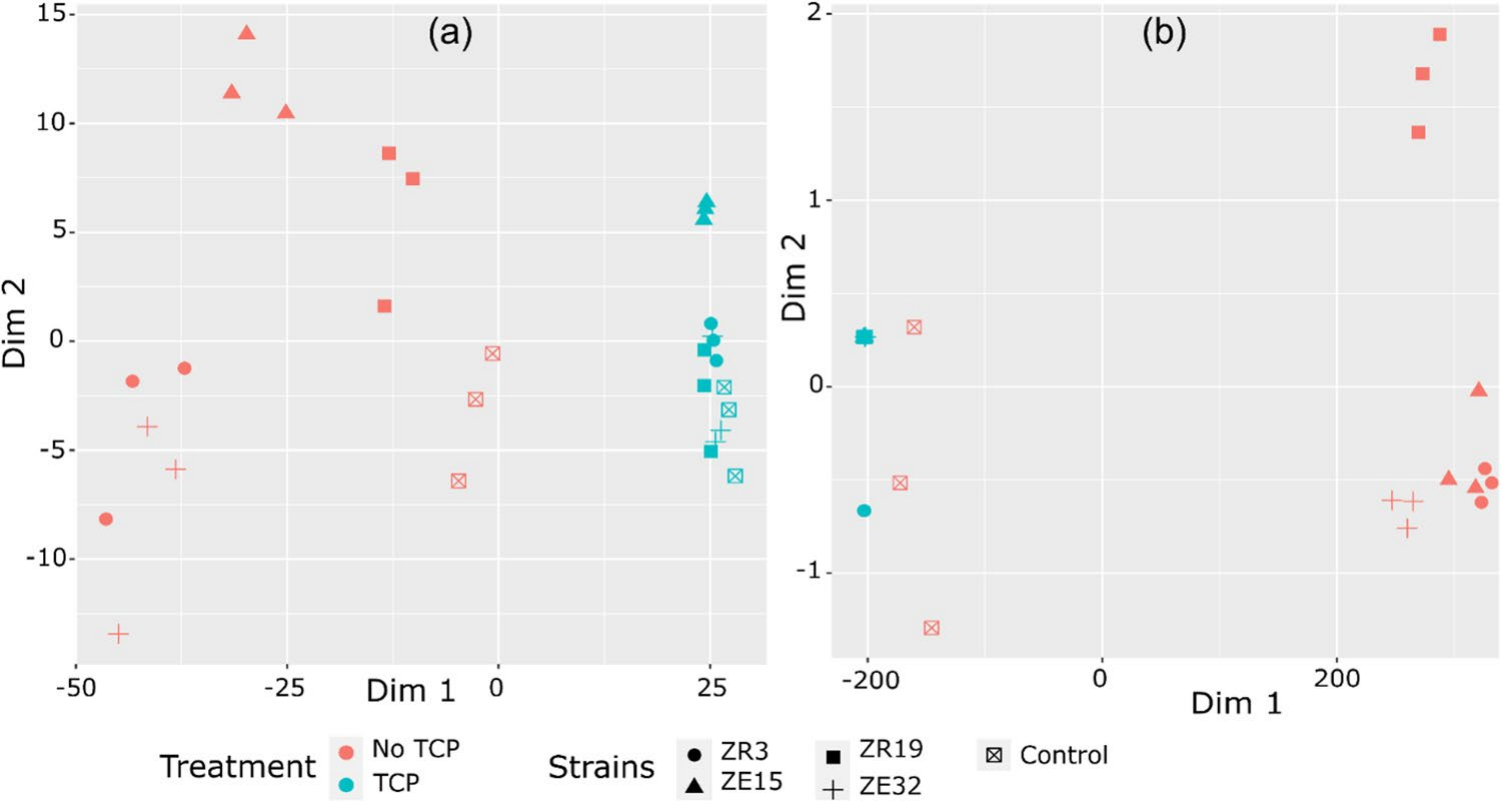
Plotting in a multidimensional scaling (MDS) format illustrates the bacterial metabolite production in Pickovskay’s broth culture. The visualization employs color differentiation based on the identification technique: Flame Ionization Detector-GC-FID analysis (**a**, on the left) and Ion Exclusion Chromatography-GC-IEC (**b**, on the right). The colors represent distinct treatments—red for the absence of tricalcium phosphate and blue for the presence of tricalcium phosphate. The shapes correspond to different bacterial strains. The visible separation between the treatments highlights the considerable impact of tricalcium phosphate (TCP) on the quantity of bacterial organic acids

Three (3) non-volatile organic acids (Supplementary Fig. 2) and eighteen (18) volatile organic acids (Supplementary Fig. 3) were determined in Pickovaskay’s broth culture in either presence or absence of insoluble P. The contribution of individual organic acids to the total production was assessed, revealing that lactic acid, propionic acid, and arachidic acid collectively accounted for over 90% of the cumulative organic acid yield by the tested strains. This observation was consistent across the range of strains studied. Furthermore, employing multidimensional scaling for organic acids highlighted a pronounced increase in organic acid accumulation in the existence of insoluble phosphorus (Fig. 3). Notably, strain ZE15 exhibited the highest production of organic acids and was distinctly separated from other strains under these conditions.

The behavior of the strains regarding organic acid secretion in the existence or absence of Ca_3_(PO_4_)_2_ (tricalcium phosphate) was explored, with particular attention to differences in production levels. Apart from isomargaric acid (Table 1), significant increases in the release of organic acids were observed when Ca_3_(PO_4_)_2_ was present. Notably, lactic acid, acetic acid, and succinic acid were commonly produced by most strains in existence of Ca_3_(PO_4_)_2_, excluding strain ZR19. Strain ZR3 was the sole producer of acetic acid, while none of the tested strains exhibited succinic acid production without Ca_3_(PO_4_)_2_. Strain ZR3 displayed the highest yield of lactic acid (533 ± 2.59 µg mL^−1^). The next strain which performed better was ZE15 (518 ± 8.14 µg mL^−1^) when cultivated in media supplemented with insoluble phosphorus. The leading producers of acetic acid and succinic acid among the tested strains were ZR3 (2 ± 0.04 µg mL^−1^) and ZR19 (2 ± 0.38 µg mL^−1^), respectively.

**Table 1:** Organic acids produced by tested bacterial strains in presence and absence of Ca_3_(PO_4_)_2_

Treatments		Lactic acid	Acetic acid	Succinic acid	Propionic acid	Butyric acid	Valeric acid	Caproic acid	Capric acid	Lauric acid	Isopalmitic acid	Palmitic acid
Control	TCP	46.9e	0.9	ND	30.8d	1.96b	1.99ab	1.58bcd	8.11de	3.63c	ND	0.85e
	No TCP	3.3f	ND	ND	1.32e	1.44c	1.50c	1.09e	8.17e	3.94b	ND	1.48d
ZE15	TCP	518b	1.8a	1.0b	53.7b	2.32a	2.15ab	1.88d	8.36abcd	4.48a	0.80c	1.55d
	No TCP	3.3f	ND	ND	1.95e	2.08ab	2.07ab	1.50 cd	8.19cde	4.01b	ND	1.52d
ZE32	TCP	464d	1.9a	0.8b	68.0a	2.26ab	2.30a	2.49a	8.52a	4.41a	0.95b	3.99a
	No TCP	4.3f	ND	ND	2.23e	1.47c	1.81bc	1.79bcd	8.23ce	3.96b	ND	1.61d
ZR3	TCP	533a	2.0a	1.1b	69.5a	2.35a	2.14ab	1.85bc	8.55a	4.32a	ND	3.65a
	No TCP	34de	1.0b	ND	2.13e	2.21ab	2.07ab	1.72bcd	8.27bcde	3.94b	ND	2.11c
ZR19	TCP	483c	ND	2.0a	38.0c	2.09ab	2.13ab	1.87bc	8.50ab	4.36a	1.60a	2.72b
	No TCP	3.3f	ND	ND	3.60e	1.50c	2.02ab	1.46de	8.42abc	3.99b	ND	2.29c
LSD (*p* ≤ 0.05)		12.77	0.4863	0.3598	4.5960	0.3029	0.3706	0.3804	0.2440	0.2832	0.1180	0.4216
Treatments		Isomargaric acid	Isostearic acid	Stearic acid	Elaidic acid	Linolic acid	Arachidic acid	Eicosenoic acid	Dacasenoic acid	Erurcic acid	Tatradecasenoic acid	
Control	TCP	1.30c	ND	1.89 fg	ND	2.72d	42.8 cd	1.0d	ND	2.23 cd	ND	
	No TCP	1.82a	ND	1.29 h	ND	2.59d	36.4e	ND	ND	1.47d	ND	
ZE15	TCP	1.35bc	4.27a	2.52de	ND	9.42a	55.3a	15.9a	ND	3.40bc	ND	
	No TCP	1.91a	ND	1.63gh	2.08a	4.76b	49.1b	ND	ND	2.30 cd	ND	
ZE32	TCP	1.52b	ND	3.49c	ND	3.62c	48.0bc	3.2c	2.46d	16.38a	ND	
	No TCP	1.88a	ND	1.67gh	1.06b	3.03 cd	38.8de	ND	ND	4.56b	ND	
ZR3	TCP	1.39bc	ND	4.22b	1.02b	3.19 cd	51.3ab	ND	4.88a	2.50 cd	2.74a	
	No TCP	1.87a	ND	2.23ef	ND	2.98 cd	41.9cde	ND	2.69b	1.86d	ND	
ZR19	TCP	1.38bc	ND	4.83a	ND	3.31 cd	53.5ab	5.4b	1.81c	2.62 cd	1.55b	
	No TCP	1.85a	ND	2.91d	ND	2.89 cd	38.7de	ND	ND	1.88d	ND	
LSD (*p* ≤ 0.05)		0.1786	4.27	0.4025	0.1098	0.8061	6.1921	0.4062	0.3993	1.1852	0.6320	

Data are present as mean of three replicates. Means followed by the same letter(s) within a column do not differ significantly (*p* ≤ *0.05*)

*ND* not detected, *TCP* presence of tricalcium phosphate, *No TCP* absence of tricalcium phosphate

Under the influence of Ca_3_(PO_4_)_2_, strain ZR3 exhibited elevated levels of propionic acid, butyric acid, and capric acid, achieving concentrations of 69.5 ± 3.11 µg mL^−1^, 2.35 ± 0.13 µg mL^−1^, and 8.55 ± 0.06 µg mL^−1^, respectively. On the other hand, strain ZE32 demonstrated maximal detection of valeric acid, palmitic acid, caproic acid, and erucic acid, with concentrations measuring 2.30 ± 0.16 µg mL^−1^, 3.99 ± 0.07 µg mL^−1^, 2.49 ± 0.15 µg mL^−1^, and 16.38 ± 1.14 µg mL^−1^, respectively.

For strain ZE15, substantial amounts of various fatty acids were synthesized, including eicosenoic acid (15.9 ± 0.03 µg mL^−1^), lauric acid (4.48 ± 0.06 µg mL^−1^), arachidic acid (55.3 ± 1.85 µg mL^−1^), and linoleic acid (9.42 ± 0.77 µg mL^−1^). Remarkably, only strain ZE15 exhibited isosteric acid production, yielding 4.27 ± 0.02 µg mL^−1^ in existence of Ca_3_(PO_4_)_2_. Strain ZR19 was responsible for the synthesis of the most elevated concentrations of isopalmitic acid (1.60 ± 0.10 µg mL^−1^) and stearic acid (4.83 ± 0.15 µg mL^−1^). Strains ZE15 and ZE32 revealed the presence of elaidic acid in the absence of insoluble phosphorus, while tetracosenoic acid was produced by strains ZR3 and ZR19.

### Evaluation of the Strains’ Growth Diversity at Different Levels of pH

**Fig. 4:**
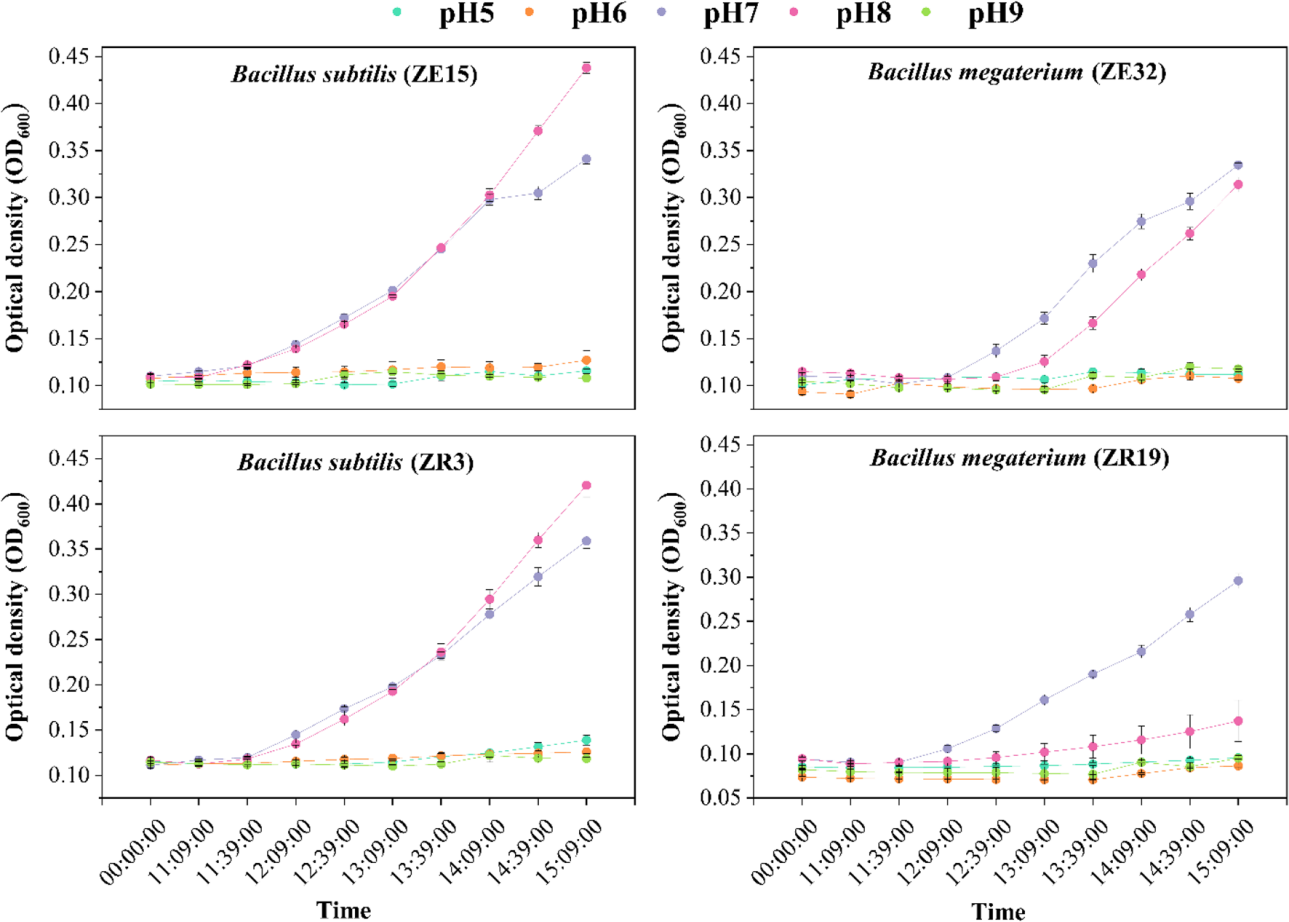
Growth of bacterial strains (optical density at 600 nm) in the pH range of 5 to 9

The outcomes of the strains’ growth in liquid culture with differing pH conditions are depicted in Fig. 4. Generally, distinct growth patterns were observed for the bacterial strains under acidic and alkaline pH conditions. When the pH decreased below 7, there was an evident reduction in growth across all strains. Strain ZE15 exhibited growth until pH 5, whereas strain ZR19 displayed growth initiation until pH 4; beyond these points, growth ceased. Strains ZR3 and ZE32 consistently demonstrated growth as the pH levels gradually decreased to 3. Strains ZE32 and ZR3 exhibited consistent growth as the pH decreased to 3. Conversely, under alkaline pH conditions, all tested strains demonstrated the ability to initiate growth, extending up to pH 9.

### Plant Growth–Promoting Potential of Tested Strains in a Rhizobox Trial

**Fig. 5:**
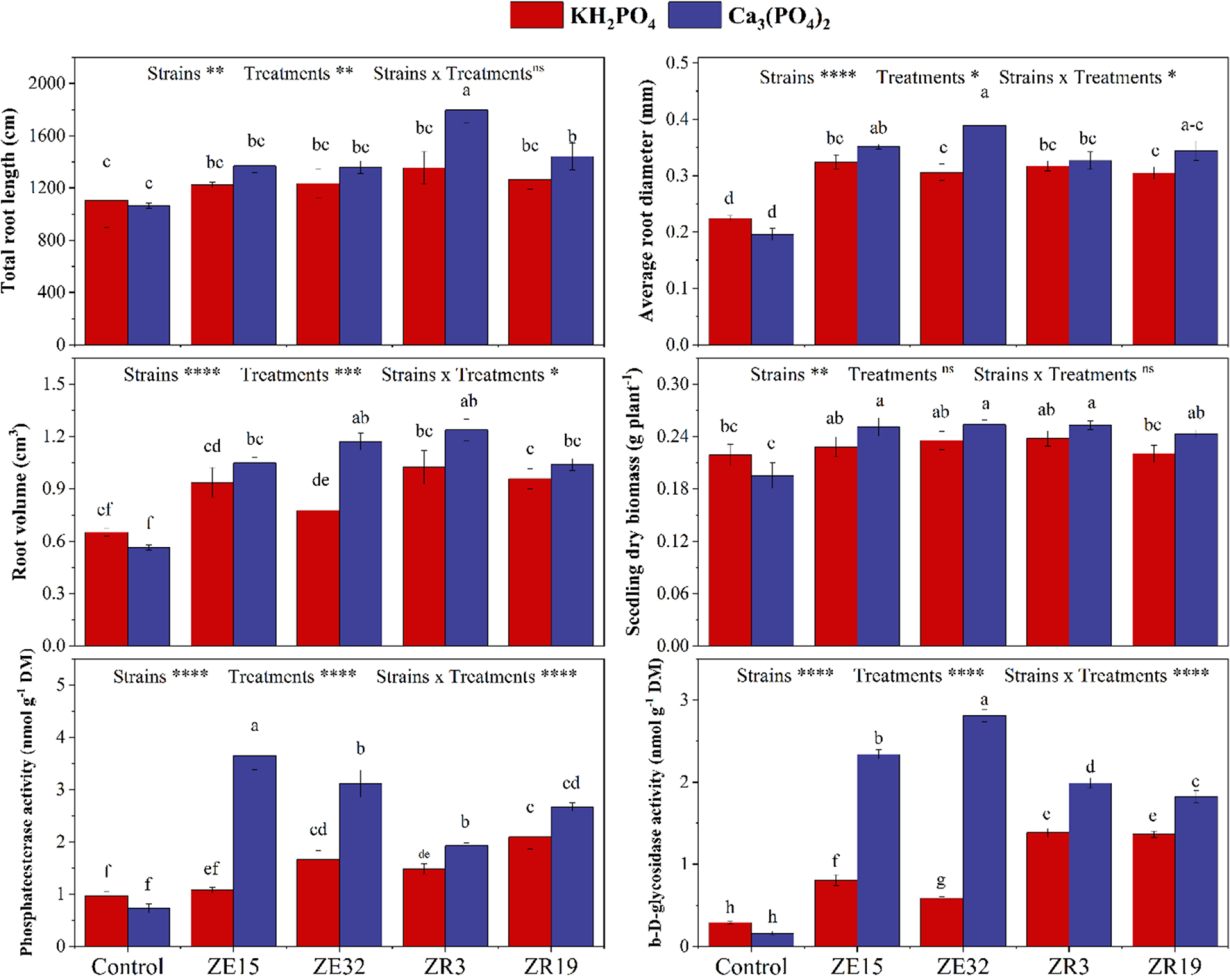
Influence of tested bacterial strains on the development of roots and the dry biomass of seedlings when exposed to insoluble (tricalcium phosphate) and soluble (potassium dihydrogen phosphate) forms of phosphorus. Bars with common letter(s) indicate no substantial variations at a significance level of 5%. *p-value below 0.05; **p-value below 0.01; ***p-value below 0.001; ****p-value below 0.0001

This investigation was conducted under the influence of two distinct phosphorous sources—one being the insoluble compound Ca_3_(PO_4_)_2_, and the other being the soluble compound KH_2_PO_4_, as illustrated in Fig. 5. In the existence of Ca_3_(PO_4_)_2_, significant variations in root length were observed among the strains. The strain ZR3 exhibited the most remarkable effect, leading to a 69% enhancement, followed by strain ZR19 which exhibited a 35% rise in root length. Conversely, when subjected to KH_2_PO_4_, no strains displayed a statistically substantial improvement in root length compared to the control.

When exposed to insoluble phosphorous (Ca_3_(PO_4_)_2_), strain ZE32 displayed the maximum root diameter (0.389 ± 0.03 mm), whereas ZR3 exhibited the highest dry biomass (0.253 ± 0.005 g plant^−1^) and root volume (1.24 ± 0.05 cm^3^). Notably, strains ZR19 and ZE15 exhibited elevated root volume, root diameter, and dry biomass compared to the control group in this condition. Subsequently, in the existence of soluble phosphorous (KH_2_PO_4_), strains ZR19 and ZE15 did not induce any significant change in dry biomass when compared to the control.

### The Comparative Effect of Insoluble P on Phosphate Esterase and β-D-Glucosidase Activities

The assessment of phosphate esterase and β-D-glucosidase activities was conducted subsequent to the harvesting of wheat seedlings, as illustrated in Fig. 5. Among the investigated strains, namely ZE32, ZE15, ZR19, and ZR3, notable variations in phosphate esterase activity were evident upon exposure to insoluble P treatment. The strain ZE15 exhibited the most pronounced phosphate esterase activity, recording a value of 3.65 ± 0.27 nmol g^−1^ dry soil, followed by strain ZE32 with a value of 3.12 ± 0.26 nmol g^−1^ dry soil. Conversely, strain ZR19 displayed reduced phosphate esterase activity under conditions involving soluble P, demonstrating a value of 2.09 ± 0.23 nmol g^−1^ dry soil. Incorporating insoluble P treatment led to elevated levels of β-D-glucosidase activity compared to situations involving soluble P. Particularly, strain ZE32 exhibited the most substantial effect on β-D-glucosidase activity (2.81 ± 0.07 nmol g^−1^ dry soil); after that, the strains ZR19 and ZE15 performed better which showed β-D-glucosidase activity as 1.82 ± 0.07 and 2.34 ± 0.05, nmol g^−1^ dry soil, respectively. Controls that were not subjected to inoculation and were treated with insoluble P displayed the lowest levels of both phosphate esterase and β-D-glucosidase activities among all experimental conditions.

### Relationship Between Microbial Exoenzyme Activities and Seedling Growth

**Fig. 6:**
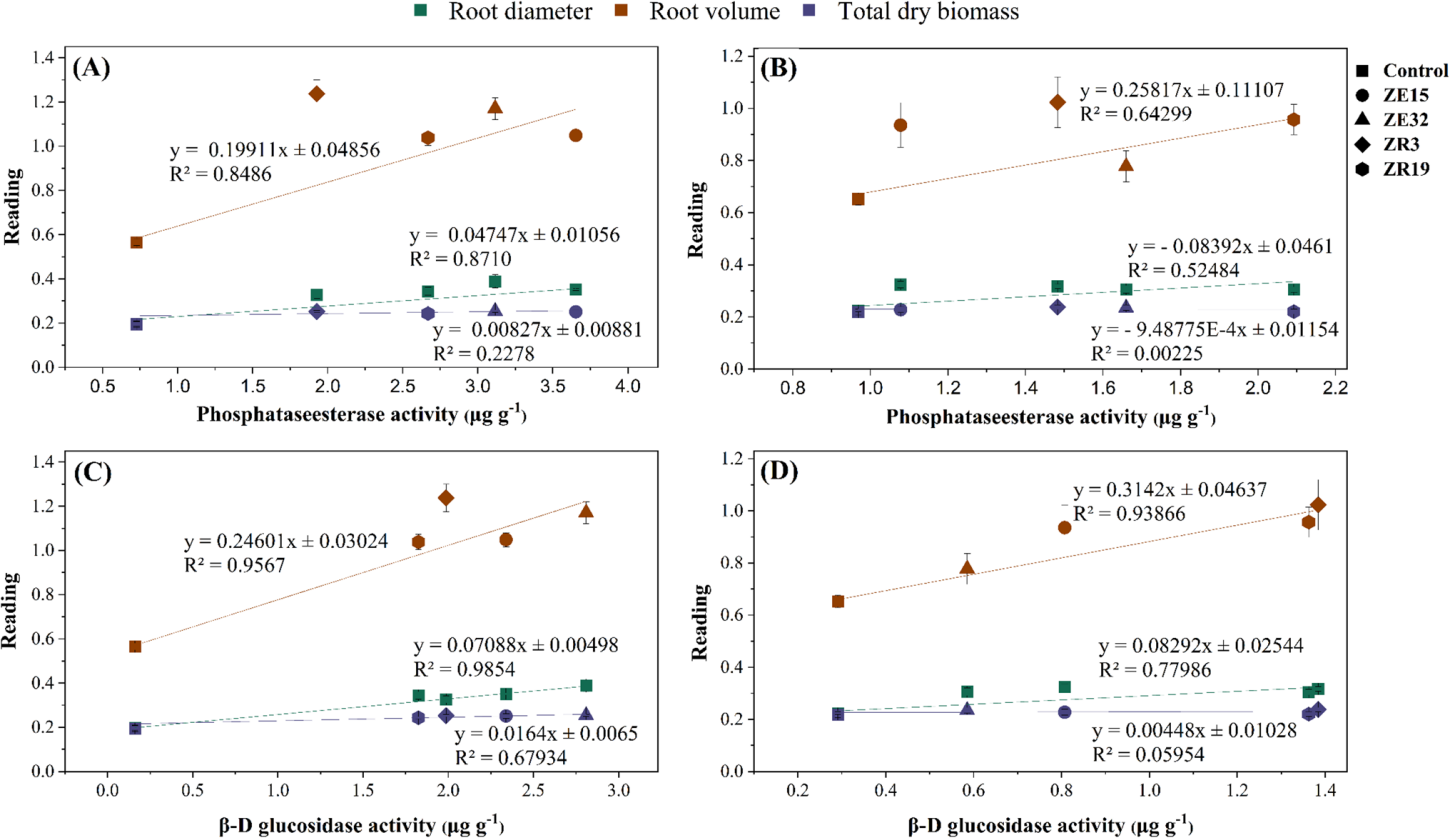
Regression analysis of growth room trial. **A** and **C** The relationship of phosphate esterase activity and β-D glucosidase activity with seedling growth in the presence of insoluble phosphorus, respectively. **B** and **D** The relationship of phosphate esterase activity and β-D glucosidase activity with seedling growth in the presence of soluble P, respectively

The data given in Fig. 6 described that the soil exoenzyme activities such as phosphate esterase and β-D glucosidase are closely related to the seedling growth. The analysis revealed a higher coefficient of determination (*R*^2^) between phosphate esterase activity and various parameters of root growth, including root volume, root diameter, and total dry biomass, indicating a positive correlation between bacterial enzyme activity and root development in the presence of insoluble phosphorus (P). The *R*^2^ values for these parameters were 0.85 (*p* < 0.0000), 0.87 (*p* < 0.0000), and 0.23 (*p* < 0.01), respectively. Conversely, in the presence of soluble P, significantly lower *R*^2^ values were observed for root volume (0.64), root diameter (0.524), and total dry biomass (0.0025). The coefficient of determination (*R*^2^) also indicated a positive relationship between β-D glucosidase and seedling growth. In the presence of insoluble P, the *R*^2^ values for root volume, root diameter, and total dry biomass were 0.96, 0.98, and 0.68, respectively. The lower *R*^2^ values for root volume (0.94), root diameter (0.78), and total dry biomass (0.06) were observed in the presence of insoluble P.

## Discussion

The findings of this investigation demonstrate the bacterial strains subjected to testing for the capability to solubilize Ca_3_(PO_4_)_2_ in Pickovskaya’s broth under varying pH conditions. This solubilization process was primarily facilitated by the production of organic acids. The outcomes of this study are consistent with prior research, which has established a clear association between decreased pH levels and enhanced P solubilization. Our experimental data captured a reduction in the pH of the culture media, with strain ZE15 prompting a decline to a minimum pH of 4.58. These results correspond to the conclusions reached by Marra et al. [23], highlighting that a lowering in pH elevated P solubilization. However, it is important to highlight those specific studies have established clear associations between the pH of the cultivation medium and the solubilization of P [24].

The stationary phase of bacterial growth occurred by the sixth day of incubation, after which negligible alterations were observed. Similar patterns have been reported in previous studies, pointing to an inverse relationship between bacterial growth and culture pH over the incubation period [25]. The production of organic acids by the strains was confirmed through GC–MS analysis, which revealed that the presence of insoluble P had a substantial effect on organic acid production. These findings align with investigations by Zeng et al. [26]. Furthermore, it was documented that a positive correlation exists between the soluble P content in the soil and the secretion of organic acids [24]. In reaction to the release of root exudates, PSB (phosphate-solubilizing bacteria) moves towards the root system, thereby impacting the composition of the rhizosphere microbial community and the arrangement of the root system [27]. Phosphorus solubilization stands as the primary function within agroecosystems for phosphate-solubilizing bacteria (PSB). Nevertheless, distinct strains of PSB exhibit varying capacities for solubilizing phosphorus [13].

The in vitro experiments conducted in this study showed notable levels of solubilized P content within Pickovskay’s broth culture. Maximum solubilized P content was achieved by the tested strains on the sixth day of incubation, sustaining a consistent level thereafter. Similar observations have been documented in existing literature, with studies reporting the stability of soluble P concentrations over time [28]. This increase in solubilized P content is attributed to the organic acids produced by the bacteria [29]. Additionally, it has been documented that the activity of alkaline phosphatase intensifies in response to the incorporation of organic matter [30]. This organic matter in the rhizosphere serves as a source of energy for microbial growth, thereby elevating phosphatase activity and encouraging the secretion of organic acids [31]. The scientific literature demonstrates that the regulation of phosphatase production in the soil microbiome is intricately linked to the availability of inorganic phosphorus and nitrogen. As shown by previous studies, the introduction of nitrogen has been observed to enhance phosphatase activity [32]. Conversely, the presence of inorganic phosphorus has been found to inhibit the synthesis and function of phosphatases, primarily due to the operation of a negative feedback mechanism [33].

The examined strains showed adaptability to grow across a range of pH levels, encompassing both acidic and alkaline conditions. The research outcomes emphasize that the highest bacterial growth was detected under neutral and alkaline pH conditions, while growth was restricted in acidic pH environments. These findings are parallel to the outcomes reported by Arindam et al. [34], which highlighted optimal bacterial growth at neutral pH levels. *Bacilli* strains exhibit the capacity to grow over a wide pH range (3–10), attaining peak growth performance at pH 7. Likewise, other investigations have identified the capacity of tested isolates to survive within pH ranges of 6 to 8.5, with approximately 83% demonstrating growth viability at pH 10 [35].

The assessed strains also manifested a significant influence on seedling growth in rhizobox trials conducted under axenic conditions. The presence of insoluble P was linked to a noteworthy increase in wheat seedling growth, surpassing the growth observed in the un-inoculated control. Conversely, the introduction of soluble P yielded a marginal impact on growth promotion by tested strains. This observation is linked with earlier studies that have highlighted the role of rhizobacteria in producing organic acids when exposed to insoluble P, thereby improving the accessibility of P for plant absorption which ultimately resulted in plant growth stimulation [36]. Additionally, plant growth promotion was positively influenced by the potential application of phosphate-solubilizing bacteria (PSB) [37]. Furthermore, microbial growth in the rhizosphere is negatively impacted by the availability of soluble P [38]. Within our investigation, the utilization of plant-associated bacteria has yielded improvements in root growth, including root length, diameter, and volume. This coincides with the findings of Setiawati et al. [39], who have reported that the incorporation of organic acids and PGPR leads to increased soluble P content in the soil, directly influencing root growth. Similar conclusions have been reached by Li et al. [40]. The relationship between the secretion of organic acids in the rhizosphere and solubilized P content in the soil was expressed by Naher et al. [41]. Our investigation has confirmed this association by demonstrating a connection between the soil’s soluble P content and the growth of plant roots. Furthermore, the inoculation of rhizobacteria has been associated with the synthesis of phytohormones that contributes to plant growth [42]. In line with this, Mumtaz et al. [43] discovered that *Paenibacillus* sp. has the capability to produce organic acids, which in turn promotes the growth of maize crops. Numerous investigations have provided evidence of PGPR application resulting in improved development, productivity, and grain quality in cereal crops [44]. To gain deeper insights into the relationship between P solubilization and the promotion of plant growth, measurements of soil exo-enzyme activities were undertaken. The findings have highlighted variances in soil exo-enzyme activities within the rhizosphere when comparing the presence of soluble and insoluble phosphorus. The tested strains have displayed increased phosphate esterase and ß-D-glucosidase activity when exposed to insoluble P, in contrast to when soluble P was present. Microbial exo-enzymes have been recognized as integral indicators of soil biochemical processes and play a pivotal role in these reactions [45]. The impact of inorganic phosphorus (P) application on phosphatase activity has been well-documented in the literature [33]. Elevated levels of phosphate solubilization activity were observed in the absence of available phosphorus [46]. It was highlighted that under P-scarce conditions, plant roots have the capacity to release acid phosphatase [47]. This release may lead to the potential improvement of phosphate solubilization activities.

In the current study, our findings have indicated an increase in ß-D-glucosidase activity with the application of *Bacillus* strains. The glycosidase enzyme plays a crucial role in breaking down chitin and other polymers with β-1,4-linked glucosamine, releasing individual N-acetyl glucosamine units [48]. This enzymatic hydrolysis process is well-known for its significance in carbon (C) and nitrogen (N) cycling [49]. Thus, the increase in ß-D-glucosidase activity might be related to phosphate solubilization as it is consistent with existing literature, which has suggested that nitrogen (N) fertilization can stimulate the activity of extracellular enzymes involved in phosphorus cycling. A sufficient supply of nitrogen appears to support soil microbial communities in producing a greater quantity of extracellular enzymes associated with hydrolytic phosphorus acquisition [50]. Furthermore, the bacterial colony counts have shown a higher number of colonies in the rhizosphere of wheat while inoculated with strains ZE15 and ZE32. The greater abundance of bacterial colonies of endophytic strains ZE15 and ZE32 may be attributed to their close relationship with the roots, as prior research has indicated their superior colonization ability compared to rhizobacterial strains ZR3 and ZR19 [16].

## Conclusion

The strains, *Bacillus megaterium* ZE32 and ZR19 and *Bacillus subtilis* ZE15 and ZR3 having P solubilization capability grow at a wide pH range of 3.0 to 10.0. However, maximum bacterial growth and activities were observed at pH 7. These strains have been observed to produce various organic acids in broth culture, leading to increased levels of solubilized P. Furthermore, ß-D-glucosidase and phosphate esterase activity in the rhizosphere of wheat crops has also indicated the P-solubilizing ability of tested strains in soil. Consequently, these strains hold promising potential as bio-inoculants for addressing the issue of P deficiency. These tested strains exhibited growth-promoting effects and represent potential candidates for effective biofertilization in P-deficient soils, pending further assessment of P solubilization under varying pH conditions, genetic modifications of bacteria under P-deficient conditions, and evaluation in field trials.

### Supplementary Information

Below is the link to the electronic supplementary material.

Supplementary file1 (DOCX 755 KB)

## Data Availability

The mean values of the required data are presented in the manuscript in the form of figures and tables, whereas raw data (replicated data in Excel) is available and will be provided at the editor’s request.

## References

[1] Matos AD, Gomes IC, Nietsche S, Xavier AA, Gomes WS, Dos Santos JA, Pereira MC (2017). Phosphate solubilization by endophytic bacteria isolated from banana trees. An Acad Bras Ciênc.

[2] Sharma SB, Sayyed RZ, Trivedi MH, Gobi TA (2013). Phosphate solubilizing microbes: sustainable approach for managing phosphorus deficiency in agricultural soils. Springer Plus.

[3] Khan MS, Zaidi A, Ahmad E (2014) Mechanism of Phosphate Solubilization and Physiological Functions of Phosphate-Solubilizing Microorganisms. *In: Phosphate Solubilizing Microorganisms, *Khan M, Zaidi A, Musarrat J (eds.). Springer, Cham, pp. 31–62. 10.1007/978-3-319-08216-5_2

[4] Mei C, Chretien RL, Amaradasa BS, He Y, Turner A, Lowman S (2021). Characterization of phosphate solubilizing bacterial endophytes and plant growth promotion in vitro and in greenhouse. Microorganisms.

[5] Amy C, Avice JC, Laval K, Bressan M (2022). Are native phosphate-solubilizing bacteria a relevant alternative to mineral fertilizations for crops? Part II: PSB inoculation enables a halving of P input and improves the microbial community in the rapeseed rhizosphere. Rhizosphere.

[6] Ameen F, AlYahya SA, AlNadhari S, Alasmari H, Alhoshani F, Wainwright M (2019). Phosphate solubilizing bacteria and fungi in desert soils: species, limitations and mechanisms. Arch Agron Soil Sci.

[7] Saeid A, Prochownik E, Dobrowolska-Iwanek J (2018). Phosphorus solubilization by Bacillus species. Molecules.

[8] Istina IN, Widiastuti H, Joy B, Antralina M (2015). Phosphate-solubilizing microbe from Saprists peat soil and their potency to enhance oil palm growth and P uptake. Procedia Food Sci.

[9] Zeng Q, Wu X, Wang J, Ding X (2017). Phosphate solubilization and gene expression of phosphate-solubilizing bacterium Burkholderia multivorans WS-FJ9 under different levels of soluble phosphate. J Microbiol Biotechnol.

[10] Elhaissoufi W, Khourchi S, Ibnyasser A, Ghoulam C, Rchiad Z, Zeroual Y, Lyamlouli K, Bargaz A (2020). Phosphate solubilizing rhizobacteria could have a stronger influence on wheat root traits and aboveground physiology than rhizosphere P solubilization. Front Plant Sci.

[11] Alori ET, Glick BR, Babalola OO (2017). Microbial phosphorus solubilization and its potential for use in sustainable agriculture. Front Microbiol.

[12] Hussain A, Ahmad M, Mumtaz MZ, Ali S, Sarfraz R, Naveed M, Jamil M, Damalas CA (2020). Integrated application of organic amendments with Alcaligenes sp. AZ9 improves nutrient uptake and yield of maize (Zea mays). J Plant Growth Regul.

[13] Manzoor M, Abbasi MK, Sultan T (2017). Isolation of phosphate solubilizing bacteria from maize rhizosphere and their potential for rock phosphate solubilization–mineralization and plant growth promotion. Geomicrobiol J.

[14] Eisenhauer N, Lanoue A, Strecker T, Scheu S, Steinauer K, Thakur MP, Mommer L (2017). Root biomass and exudates link plant diversity with soil bacterial and fungal biomass. Sci Rep.

[15] Tian J, Ge F, Zhang D, Deng S, Liu X (2021). Roles of phosphate solubilizing microorganisms from managing soil phosphorus deficiency to mediating biogeochemical P cycle. Biology.

[16] Iqbal Z, Ahmad M, Jamil M, Akhtar M (2020). Appraising the potential of integrated use of Bacillus strains for improving wheat growth. Int J Agric Biol.

[17] Clesceri LS, Greenberg AE, Eaton AD (1998) Standard Methods for the Examination of Water and Wastewater. 20th Edition, American Public Health Association, Washington DC

[18] Politz M, Lennen R, Pfleger B (2013). Quantification of bacterial fatty acids by extraction and methylation. Bio-Protoc.

[19] Hoagland DR, Arnon DI (1950) The Water-Culture Method for Growing Plants without Soil. 2nd edition. California Agricultural Experiment Station, Circular 347

[20] Hendriksen N, Creamer R, Stone D, Winding A (2016). Soil exo-enzyme activities across Europe—the influence of climate, land-use and soil properties. Appl Soil Ecol.

[21] Steele R, Torrey J, Dickeys D (1997) Multiple comparisons. Principles and procedures of statistics–a biometrical approach. McGraw Hill, New York

[22] Giorgi FM, Ceraolo C, Mercatelli D (2022). The R language: an engine for bioinformatics and data science. Life.

[23] Marra LM, Oliveira-Longatti SMd, Soares CR, Lima JMd, Olivares FL, Moreira F (2015). Initial pH of medium affects organic acids production but do not affect phosphate solubilization. Braz J Microbiol.

[24] Mihalache G, Mihasan M, Zamfirache MM, Stefan M, Raus L (2018). Phosphate solubilizing bacteria from runner bean rhizosphere and their mechanism of action. Rom Biotechnol Lett.

[25] Wang Z, Xu G, Ma P, Lin Y, Yang X, Cao C (2017). Isolation and characterization of a phosphorus-solubilizing bacterium from rhizosphere soils and its colonization of chinese cabbage (Brassica campestris ssp. chinensis). Front Microbiol.

[26] Zeng Q, Wu X, Wen X (2016). Effects of soluble phosphate on phosphate-solubilizing characteristics and expression of gcd gene in Pseudomonas frederiksbergensis JW-SD2. C Curr Microbiol.

[27] Al-Ali A, Deravel J, Krier F, Béchet M, Ongena M, Jacques P (2018). Biofilm formation is determinant in tomato rhizosphere colonization by Bacillus velezensis FZB42. Environ Sci Pollut Res.

[28] Li X, Luo L, Yang J, Li B, Yuan H (2015). Mechanisms for solubilization of various insoluble phosphates and activation of immobilized phosphates in different soils by an efficient and salinity-tolerant Aspergillus niger strain An2. Appl Biochem Biotechnol.

[29] Zuluaga MYA, de Oliveira ALM, Valentinuzzi F, Jayme NS, Monterisi S, Fattorini R, Cesco S, Pii Y (2023). An insight into the role of the organic acids produced by Enterobacter sp. strain 15S in solubilizing tricalcium phosphate: in situ study on cucumber. BMC Microbiol.

[30] Yang L, Bian X, Yang R, Zhou C, Tang B (2018). Assessment of organic amendments for improving coastal saline soil. Land Degrad Dev.

[31] Sindhu SS, Sehrawat A, Glick BR (2022). The involvement of organic acids in soil fertility, plant health and environment sustainability. Arch Microbiol.

[32] Heuck C, Smolka G, Whalen ED, Frey S, Gundersen P, Moldan F, Fernandez IJ, Spohn M (2018). Effects of long-term nitrogen addition on phosphorus cycling in organic soil horizons of temperate forests. Biogeochem.

[33] Marklein AR, Houlton BZ (2012). Nitrogen inputs accelerate phosphorus cycling rates across a wide variety of terrestrial ecosystems. New Phytol.

[34] Arindam C, Mala RH, Roshni R, Mohini J, Rashmi Y, Siddalingeshwara K, Pramod T (2014). Isolation and characterization of potential plant growth promoting rhizobacteria from non-rhizospheric soil. Int j curr microbiol appl sci.

[35] Ahmad M, Ahmad I, Hilger TH, Nadeem SM, Akhtar MF, Jamil M, Hussain A, Zahir ZA (2018). Preliminary study on phosphate solubilizing Bacillus subtilis strain Q3 and Paenibacillus sp. strain Q6 for improving cotton growth under alkaline conditions. PeerJ.

[36] Woyessa D, Assefa F (2011). Diversity and plant growth promoting proerties of rhizobacteria isolated from tef (Eragrostis tef). Ethiop J Educ sci.

[37] Sharon J, Hathwaik L, Glenn G, Imam S, Lee C (2016). Isolation of efficient phosphate solubilizing bacteria capable of enhancing tomato plant growth. J Soil Sci Plant Nutr.

[38] Zhang T, Hu F, Ma L (2019). Phosphate-solubilizing bacteria from safflower rhizosphere and their effect on seedling growth. Open Life Sci.

[39] Setiawati TC, Erwin D, Mandala M, Hidayatulah A (2022) Use of Bacillus as a Plant Growth-Promoting Rhizobacteria to Improve Phosphate and Potassium Availability in Acidic and Saline Soils. KnE Life Sci 7(3):541–558. 10.18502/kls.v7i3.11160

[40] Li H, Qiu Y, Yao T, Ma Y, Zhang H, Yang X (2020). Effects of PGPR microbial inoculants on the growth and soil properties of Avena sativa, Medicago sativa, and Cucumis sativus seedlings. Soil Tillage Res.

[41] Naher UA, Panhwar QA, Othman R, Shamshuddin J, Ismail MR, Zhou E (2018). Proteomic study on growth promotion of PGPR inoculated aerobic rice (Oryza sativa L.) cultivar MR219-9. Pak J Bot.

[42] Khan N, Bano A, Ali S, Babar MA (2020). Crosstalk amongst phytohormones from planta and PGPR under biotic and abiotic stresses. Plant Growth Regul.

[43] Mumtaz MZ, Ahmad M, Jamil M, Hussain T (2017). Zinc solubilizing Bacillus spp. potential candidates for biofortification in maize. Microbiol Res.

[44] Hussain A, Zahir ZA, Ditta A, Tahir MU, Ahmad M, Mumtaz MZ, Hayat K, Hussain S (2019). Production and implication of bio-activated organic fertilizer enriched with zinc-solubilizing bacteria to boost up maize (Zea mays L.) production and biofortification under two cropping seasons. Agronomy.

[45] Bai X, Dippold MA, An S, Wang B, Zhang H, Loeppmann S (2021). Extracellular enzyme activity and stoichiometry: the effect of soil microbial element limitation during leaf litter decomposition. Ecol Indic.

[46] Uwituze Y, Nyiraneza J, Fraser TD, Dessureaut-Rompré J, Ziadi N, Lafond J (2022). Carbon, nitrogen, phosphorus, and extracellular soil enzyme responses to different land use. Front Soil Sci.

[47] Tadano T, Sakai H (1991). Secretion of acid phosphatase by the roots of several crop species under phosphorus-deficient conditions. Soil Sci Plant Nutr.

[48] Sinsabaugh RL, Gallo ME, Lauber C, Waldrop MP, Zak DR (2005). Extracellular enzyme activities and soil organic matter dynamics for northern hardwood forests receiving simulated nitrogen deposition. Biogeochem.

[49] Lou H, Lin M, Zeng M, Cai C, Pang Y, Yang D, Qiu X (2018). Effect of urea on the enzymatic hydrolysis of lignocellulosic substrate and its mechanism. Bioenergy Res.

[50] Jian S, Li J, Chen JI, Wang G, Mayes MA, Dzantor KE, Hui D, Luo Y (2016). Soil extracellular enzyme activities, soil carbon and nitrogen storage under nitrogen fertilization: a meta-analysis. Soil Biol Biochem.

