# Supplementary material for: Phosphate-Solubilizing Bacillus sp. Modulate Soil Exoenzyme Activities and Improve Wheat Growth
Source: Microb Ecol. 2024 Jan 16;87(1):31. doi: 10.1007/s00248-023-02340-5 (PMC10791813; doi:10.1007/s00248-023-02340-5)
Supplement: Supplementary file 1 — Supplementary file1 (DOCX 755 KB) [file 248_2023_2340_MOESM1_ESM.docx]

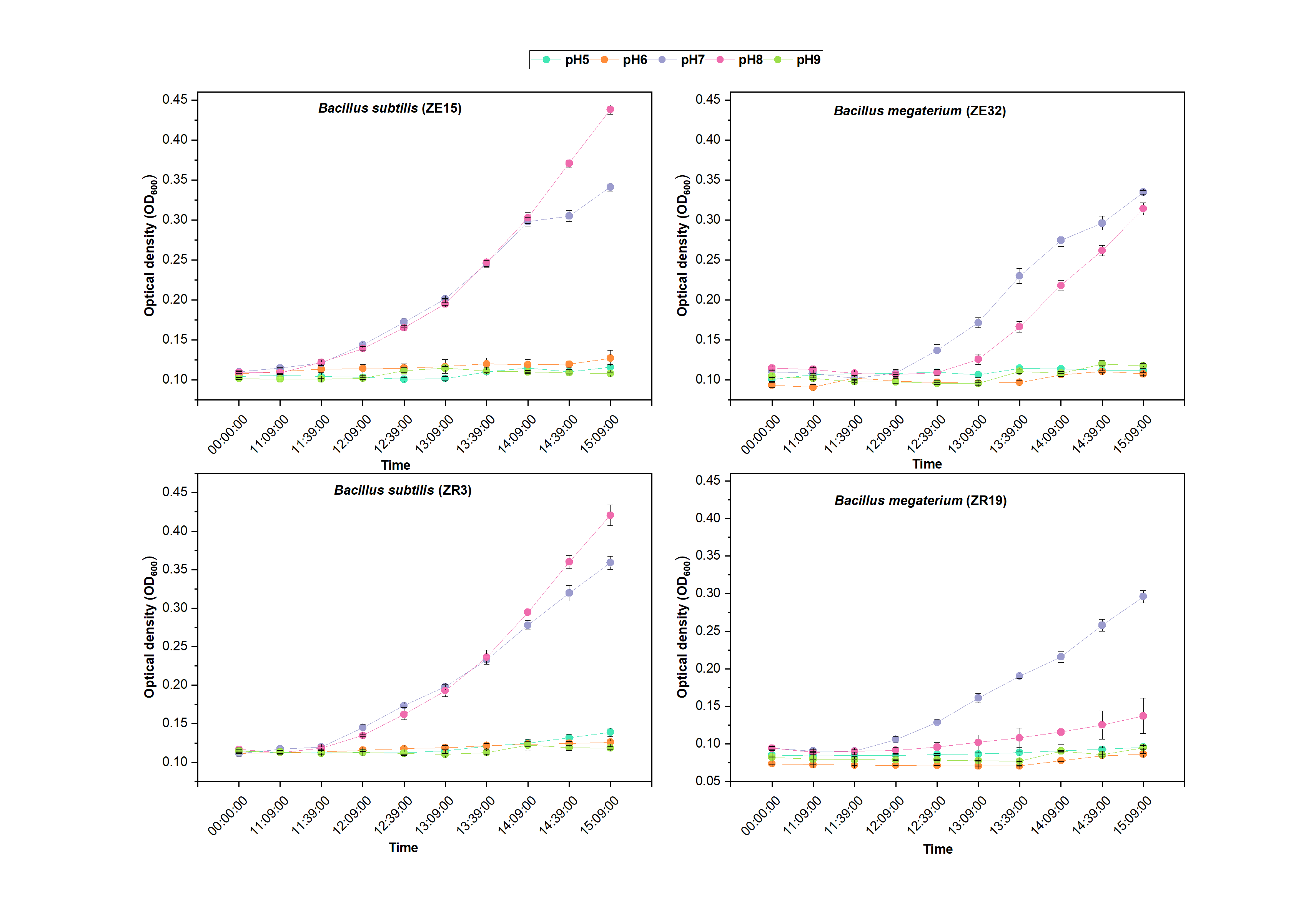


**Supplementary fig. 1:** Growth behavior of bacteria in the presence and absence of tricalcium phosphate (TCP). Bars represent the bacterial population and lines represent optical density. Data is presented in a semi-log graph; (Control) Pickovskaya's media without Ca_3_(PO_4_)_2_; (Insoluble P) Pickovskaya's media with Ca_3_(PO_4_)_2_.

**
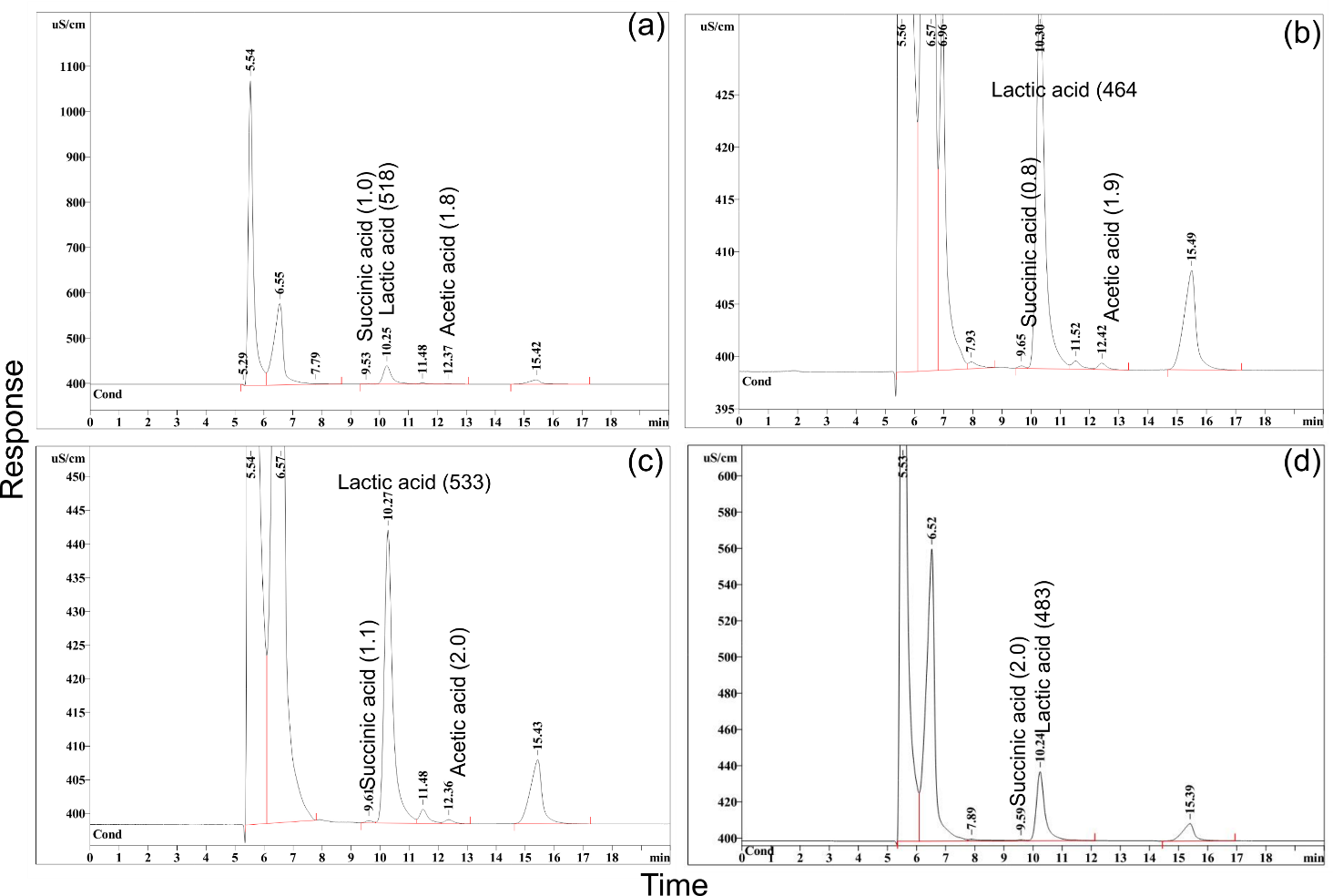
**

**Supplementary fig. 2:** Identification and quantification of organic acid produced in Pickovskay's broth culture containing tricalcium phosphate as an insoluble source. Data in parenthesis represent the concentration of acid in mg L^-1^. Ion Exclusion Chromatography; (a) Bacillus subtilis-ZE15, (b) Bacillus megaterium-ZE32, (c) Bacillus subtilis-ZR3, (d) Bacillus megaterium-ZR19.


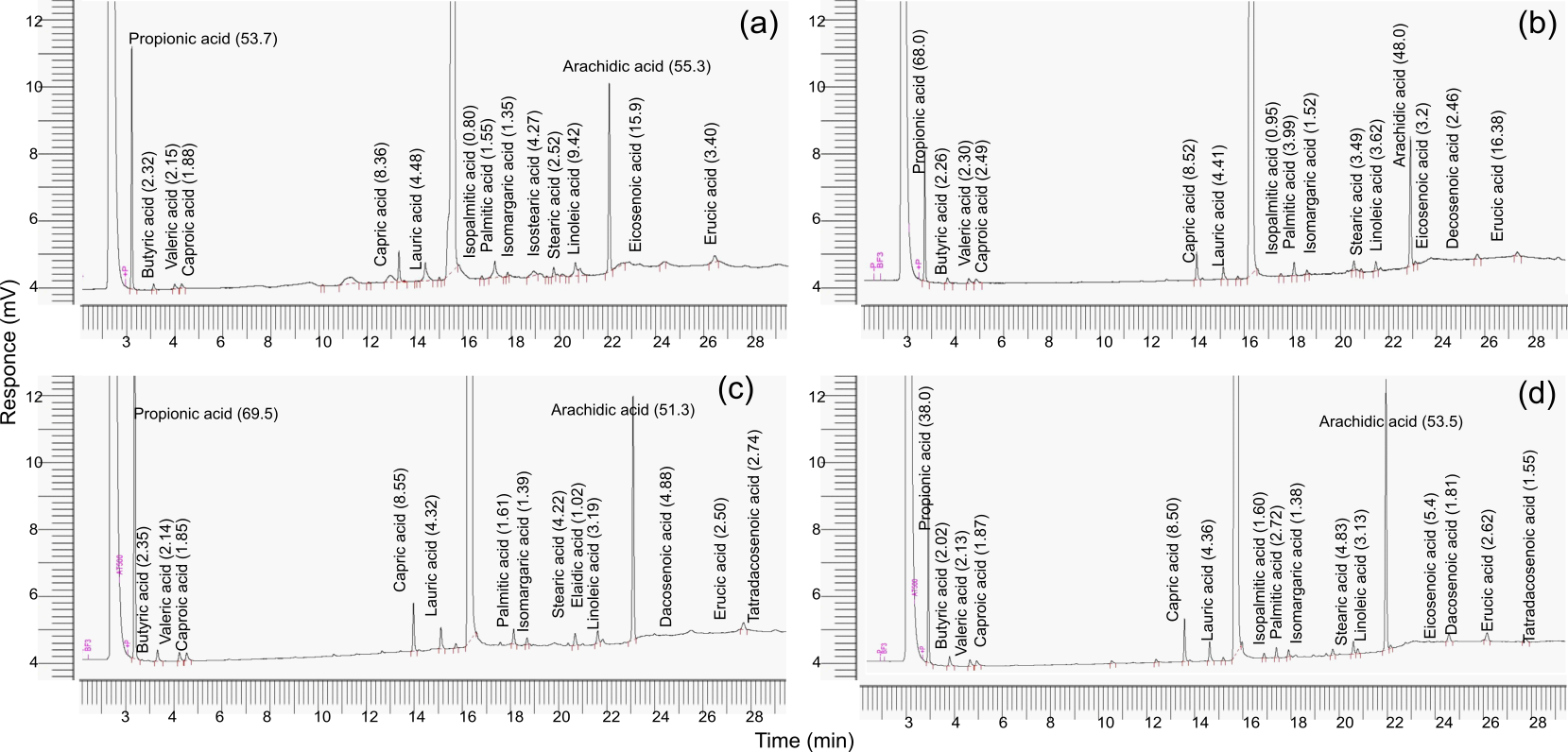


**Supplementary fig. 3:** Identification and quantification of FAME metabolites produced in Pickovskay's broth culture containing tricalcium phosphate as an insoluble source. Data in parenthesis represent the concentration of acid in mg L^-1^. GC-FID FAME analysis; (a) Bacillus subtilis-ZE15, (b) Bacillus megaterium-ZE32, (c) Bacillus subtilis-ZR3, (d) Bacillus megaterium-ZR19
